# Evaluation of the infectivity of three porcine reproductive and respiratory syndrome virus (PRRSV) variants

**DOI:** 10.1186/s13567-025-01591-z

**Published:** 2025-07-23

**Authors:** Claudio Marcello Melini, Mariana Kikuti, Montserrat Torremorell, Kimberly VanderWaal, Stephanie Rossow, Jerry Torrison, Cesar A. Corzo

**Affiliations:** 1 Department of Veterinary Population Medicine, University of Minnesota, St. Paul, MN USA; 2 Veterinary Diagnostic Laboratory, University of Minnesota, St. Paul, MN USA; 3 Longhorn Vaccines and Diagnostics LLC, Bethesda, MD USA

**Keywords:** Infectious dose, PRRSV, variant, infectivity

## Abstract

Porcine reproductive and respiratory syndrome virus (PRRSV) continues to burden the US swine industry significantly. In some instances, the virus evaded biosecurity measures, remaining viable in sufficient concentrations to cause an outbreak. Little is known about differences in infectivity among viral variants. In this study, we compared the infectivity of three PRRSV variants by estimating the median infectious dose (ID_50_) and minimum infectious dose (MID), while also characterizing nasal and rectal shedding patterns and histologic lesions. Groups of six individually housed pigs were intranasally inoculated with 2 mL of five different concentrations (10^0^ to 10^4^ TCID_50_/mL) of inoculum per variant. Blood, rectal, and nasal swabs were collected at different time points up to 28 days post-inoculation (dpi) and tested via RT-PCR. Two animals per group were euthanized on 11 dpi and tissue samples were assessed by histopathology. The estimated ID_50_ based on serum RT-PCR positive pigs at 4 dpi was 10^2.6^ TCID_50_/mL (95% CI 10^2.0^, 10^3.2^) for the L9A variant, 10^2.3^ TCID_50_/mL (95% CI 10^1.6^, 10^3.0^) for the L1A variant, and 10^1.3^ TCID_50_/mL (95% CI 10^0.4^, 10^2.2^) for variant L1C.5. No apparent differences were observed in shedding patterns among tested groups. However, the amount of lesions caused by variant L1C.5 was more extensive compared to those infected with the other two variants. Based on our findings, the L1C.5. variant required fewer infectious units to infect half of the inoculated pigs and resulted in more microscopic lesions confirming that PRRSV variants exhibit different levels of infectivity, virulence, and pathogenicity.

## Introduction

Porcine reproductive and respiratory syndrome virus (PRRSV) belongs to the family *Arteriviridae* in the order *Nidovirales,* with two distinct species classified as *Betaarterivirus europensis* (PRRSV-1) and *Betaarterivirus americense* (PRRSV-2) [1, 2]. In the United States of America (US), PRRS is considered the costliest swine disease with yearly losses in breeding and growing pig herds estimated at up to $1.2 billion USD [3]. These losses originate from premature farrowing and reproductive failure (e.g., mummies, abortions, and stillbirths), poor growth performance, respiratory disease, and increased mortality in growing pigs [4]. Upon infection, viremia can develop in pigs within 12 h and may persist up to 8 weeks, although duration of viremia is influenced by age at the time of infection [5–10]. Once pigs become viremic, infections can be prolonged, with the virus localizing in the tonsil and lymph nodes [7, 11–15]. Infected pigs can shed the virus through different routes (e.g., saliva, urine, semen, nasal secretions, feces, mammary gland secretions), which then can contaminate surfaces and serve as routes for indirect exposure to susceptible pigs [13, 15–18]. The route of infection can influence the median infection dose (ID_50_), with estimates of 10^5.3^ TCID_50_ (95% CI 10^4.6^, 10^5.9^) via the oral route, and 10^4^ TCID_50_ (95% CI 10^3^, 10^5^) via the nasal route [19].

Since its first isolation in the US in 1992, new PRRSV genetic variants have emerged, some of which have generated a high level of concern due to their perceived virulence and pathogenicity [20]. In 2020, a PRRSV variant classified as lineage (L) 1 sub-lineage C (L1C.5) emerged in southern Minnesota, simultaneously affecting farms from different pig production companies and causing significant production losses [21]. The virulence and transmissibility of L1C.5 have been assessed through comparison with four other PRRSV isolates [22], but no studies have compared the infectivity of different variants using different inoculum concentrations.

An experimental study was conducted with the objective of comparing the infectivity of three PRRSV variants by estimating their minimum infectious dose (MID) and ID_50_. In addition, nasal and rectal viral shedding patterns were characterized, along with the extent of microscopic lesions in infected pigs.

## Materials and methods

### Virus variants, dilution, and titration

Three PRRSV-2 variants representative of past and current US PRRSV epidemics were selected for the study. Viruses were classified through PRRSLoom [23] and previous methods [24–26] using ORF5 sequences. All viruses were grown and passaged on the MARC-145 [27] cell line. The first virus was an L9A variant, also known as MN-30100, which represented a prevalent virus from the mid-2000s and was propagated through six passages to a titer of 1 × 10^7.5^ TCID_50_/mL. The second variant, classified as L1A (variant unclassified), underwent nine passages and had a titer of 1 × 10^7.5^ TCID_50_/mL, representing the virus that caused an epidemic in the mid-2010s [24]. Lastly, the 2020 L1C.5 variant, with four passages and a titer of 1 × 10^6.75^ TCID_50_/mL, represented the most current epidemic.

On the day of inoculation, tenfold dilutions of each variant were prepared using EMEM to reach the desired five inoculum concentrations (i.e., 10^4^, 10^3^, 10^2^, 10^1^, 10^0^ TCID_50_/mL), obtaining eight doses from each inoculum concentration and labeling them according to their treatment. Two of the eight doses from each concentration were used as controls to assess their titer and calculate RNA copies per mL by quantitative reverse transcription polymerase chain reaction (RT-qPCR) following the manufacturer’s instructions (VetMAX^™^ PRRSV EU & NA v3.0 kit, ThermoFisher Scientific Inc., Waltham, MA, USA). Briefly, after RNA extraction from the inoculum, RT-PCR was done in triplicate to each sample by adding 12 µL of the master mix to each well and 8 µL of the standard to each appropriate well, then vortex for approximately 10 s to mix the template with the master mix, followed by centrifuge at 2000 × *g* for 10 to 15 s. Then the plate is placed in 7500 Fast reader and the following conditions were applied: 1 cycle for RT at 50 °C for 5 min, 1 cycle for Taq activation at 95 °C for 10 min, 40 cycles for denaturation at 95 °C for 3 s, and 40 cycles for annealing at 60 °C for 30 s. Controls were obtained from transcribed ORF6 RNA, the stock solution was diluted to obtain five standards (1 × 10^7^ copies/µL to 1 × 10^3^ copies/µL). Following internal standard operation procedures (SOPs) the standards values were converted to report as copies/mL and reported accordingly. Titration was done on fully grown layers of MARC-145 cells in 96-well plates, using 4 wells per dilution. The plates were incubated at 37 °C for seven days and assessed daily for cytopathic effects (CPE). The viral titer was calculated by observing the wells for CPE for up to seven dpi, following the Karber method [28].

### Experimental design

**Figure 1:**
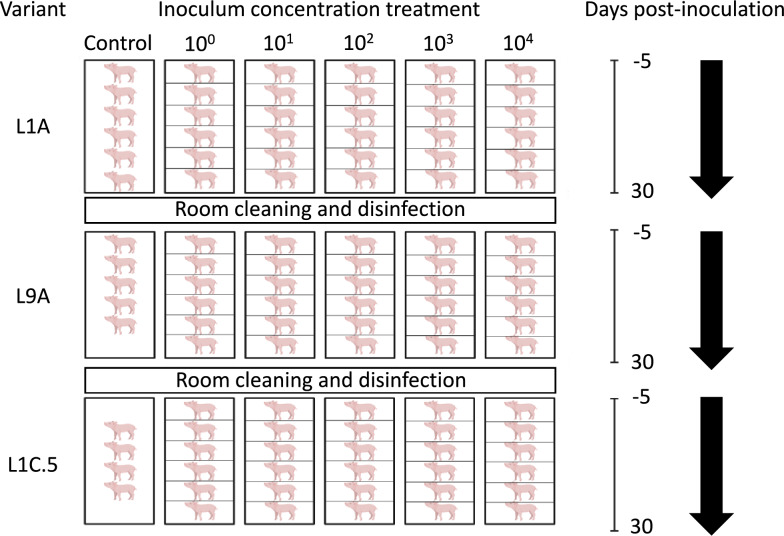
**Study design.** Order of variant group assessment and housing distribution of inoculum concentration treatments during the 36-day study duration in the isolation facility

Three groups of 34–36 4-week-old Yorkshire x Landrace breed barrows originating from a sow herd that had historically tested PRRSV-ELISA and RT-PCR negative, were established (one group for each PRRSV variant). For each variant, subgroups of six pigs were housed individually and randomly assigned to one of five PRRSV inoculum concentration treatments (i.e., 10^4^, 10^3^, 10^2^, 10^1^, 10^0^ TCID_50_/mL), along with a negative control group that was sham-inoculated with Eagle’s Minimal Essential Media (EMEM). Each subgroup comprising the combination of the different variants and inoculum concentration treatments was housed in separate rooms at the University of Minnesota Veterinary Isolation Facility (VIF) for 36 days (Figure 1). However, the experiments were conducted one variant at a time due to space limitations in the animal isolation facility. Blood samples were collected 2 days after pig arrival and acclimation (−3-day post-inoculation [dpi]) and submitted to the University of Minnesota Veterinary Diagnostic Laboratory (UMN-VDL) for antibody and virus detection using ELISA (HerdCheck^®^ PRRS IDEXX Laboratories Inc., Westbrook, ME, USA) and RT-PCR (VetMAX^™^ Thermo Fisher Scientific Inc., Waltham, MA, USA) to confirm that the pigs remained PRRSV negative before the start of the study. Regarding the PRRSV RT-PCR, first high throughput total nucleic acid extraction was done using magnetic bead technology (MagMAX^™^ CORE Nucleic Acid Purification Kit, ThermoFisher Scientific Inc., Waltham, MA, USA), then RT-PCR was performed using VetMAX^™^ PRRSV EU & NA v3.0 kit (ThermoFisher Scientific Inc., Waltham, MA, USA) following the manufacturer’s instructions. All samples at 0 dpi were collected before intranasally inoculating the pigs with 2 mL (1 mL per nostril) of the variant using a single regular 3 mL disposable syringe. The pigs in the control group were sham-inoculated first, followed by the 10^0^ -inoculum concentration treatment and then progressively higher concentrations until reaching the 10^4^ group. On 0, 1, 4, 7, 11, 16, 21, 26, and 30 dpi, blood, nasal and rectal swabs were collected to assess viremia and viral shedding. The samples were submitted to the UMN VDL and tested for PRRSV by RT-PCR. Pigs were always sampled in the same order, starting with the pen farthest from the room’s entry and then proceeding toward the front. Each sample was individually labeled, placed in resealable bags, and refrigerated at 4 °C until processing. On 11 dpi, two pigs were randomly selected from each room to be euthanized using a lethal dose of pentobarbital through the jugular vein. The remaining pigs were euthanized using the same method at 30 dpi. During necropsy, two sets of each tissue (e.g., lung, brain, lymph node, liver) samples were collected, placed in 10% neutral buffered formalin, and submitted to the UMN VDL for histopathologic assessment by a board-certified pathologist who was blinded to the inoculum concentrations and variants.

### Housing and handling

Upon arrival at the UMN VIF, ear-tagged pigs were housed in individual pens and allowed to acclimate for 5 days. Pigs had no direct contact with each other as a 1-m-high plastic divider was used to keep them separated. Each pig had ad libitum water and feed.

After inoculation, all personnel (including personnel sampling, feeding and handling pigs) were asked to shower and change clothes between rooms. Upon inoculation, personnel movement between rooms was only allowed to occur in an inoculum concentration group ascendant manner, that is, from the negative control room towards the lowest concentration treatment and continuing continued sequentially to higher concentration rooms, without returning to any previously visited room.

All personnel were asked to wear clean clothes, gloves, and a mask and, once in the ante-room, personnel changed into room-specific coveralls and boots. If pig handling was needed, personnel wore a disposable plastic gown, mask, and a fresh pair of gloves for each pig to avoid indirect transmission of the virus.

### Sample management and diagnostics

Blood samples were centrifuged at 1500 x *g* at a temperature of 4 °C for 10 min, producing two 500 µL serum aliquots. Each nasal and rectal swab was placed in 2 mL of DMEM, vortexed for 15 s, and divided into two 1 mL aliquots. To blind the UMN VDL technicians and diagnostician, each sample was relabeled with a new identification code, ensuring they were unaware of inoculum concentration and variant group. Serum and swab samples were submitted for PRRSV RT-PCR. Infection due to inoculation was defined as any pig that tested positive on dpi 4.

### Statistical analyses

To calculate the ID_50_, a generalized linear model with a probit link function was used from the “MASS” package from R statistical software [29, 30]. The model was derived from the median effective dose, a method typically used to assess the relationship between a dose and the proportion of individuals who survive [31]. In this study, we calculated the proportion of individuals that tested RT-PCR positive for PRRSV on dpi 4 (including the control treatment) as: ${\text{ID}} \left( \rho \right) = \frac{{g\left( \rho \right) - \beta_{0} }}{{\beta_{1} }}$, where ${\text{ID}} \left( \rho \right)$ is the infectious dose of any proportion of the population; *g* is the probit link function, to fit the model; $\rho$ is the proportion of the population, set to 0.5; $\beta_{0}$ is the intercept; $\beta_{1}$ represents the inoculum concentration treatment. Results were expressed in TCID_50_/mL, and for each ID_50,_ the respective 95% confidence interval (CI) was calculated. The MID for each variant was determined using the lowest inoculum concentration treatment with at least 1 positive pig. Fisher’s Exact test was used on the frequency of positive pigs at 4 dpi for each concentration treatment and for the whole PRRS variant groups to assess differences between them using fisher.test from the R statistical software “stats” package [30]. Viral shedding patterns were analyzed using descriptive statistics incorporating only those pigs that tested RT-PCR positive at 4 dpi by serum. Similarly, histopathological lesions were assessed using descriptive statistics.

## Results

All pigs tested antibody and virus negative for PRRSV on −3 dpi, and by RT-PCR again on 0 dpi, confirming their PRRSV-free status prior to inoculation. Control groups remained PRRSV-negative throughout the entire study. Titration and RT-qPCR results for each variant inoculum are summarized in Table 1, showing in the latter that the RNA copies did not always match between dilution groups of the variants, probably due to a characteristic of the specific virus adaptation and replication time. On 4 dpi the number of RT-PCR-positive animals for the L1C.5 variant was 26 out of 30, for the L1A variant it was 19 out of 30, and for the L9A variant it was 15 out of 30. Fisher’s Exact test showed that overall, there is no statistical difference between L9A and L1A viruses, but there was statistical difference between L9A and L1C.5 variant (*p*-value 0.004) and L1A and L1C.5 variant (*p*-value 0.039). But there was no statistical difference between the three viruses by each treatment group (Table 2).

**Table 1:** **Virus titration and RT-qPCR (estimated ORF6 RNA copies/mL) of the tenfold dilutions used to inoculate the pigs and the number of RT-PCR-positive pigs at 4 days post-inoculation (dpi)**

PRRSV variant	Concentration treatment	Titer (log_10_ TCID_50_/mL)	ORF6 RNA copies/mL	RT-PCR-positive pigs at 4 dpi
L9A	10^0^	NCPE*	3.13 × 10^5^	0/6
	10^1^	10^1.8^	2.99 × 10^6^	1/6
	10^2^	10^2.3^	3.37 × 10^7^	4/6
	10^3^	10^3.5^	1.45 × 10^8^	4/6
	10^4^	10^4.8^	2.08 × 10^9^	6/6
L1A	10^0^	NCPE*	4.23 × 10^6^	2/6
	10^1^	10^2.2^	3.11 × 10^7^	2/6
	10^2^	10^2.5^	1.94 × 10^8^	3/6
	10^3^	10^3.8^	3.90 × 10^9^	6/6
	10^4^	10^5.2^	3.98 × 10^10^	6/6
L1C.5	10^0^	NCPE*	7.64 × 10^4^	4/6
	10^1^	10^1.8^	5.73 × 10^5^	5/6
	10^2^	10^2.5^	5.36 × 10^6^	5/6
	10^3^	10^3.5^	4.42 × 0^7^	6/6
	10^4^	10^4.3^	4.36 × 10^8^	6/6

*No cytopathogenic effect (CPE), with a detection limit of 10^1.5^ TCID_50_/mL.

**Table 2:** **Fisher’s exact test of number of positive pigs at 4 days after inoculation between variants**

Concentration treatment group	PRRSV variant 1	RT-PCR positive pigs at 4 dpi	PRRSV variant 2	RT-PCR positive pigs at 4 dpi	Fisher’s exact test *p*-value
Overall					
	L9A	15/30	L1A	18/30	0.604
	L9A	15/30	L1C.5	26/30	0.004*
	L1A	18/30	L1C.5	26/30	0.039*
10^0^					
	L9A	0/6	L1A	2/6	0.454
	L9A	0/6	L1C.5	4/6	0.060
	L1A	2/6	L1C.5	4/6	0.567
10^1^					
	L9A	1/6	L1A	2/6	1.000
	L9A	1/6	L1C.5	5/6	0.080
	L1A	2/6	L1C.5	5/6	0.242
10^2^					
	L9A	4/6	L1A	3/6	1.000
	L9A	4/6	L1C.5	5/6	1.000
	L1A	3/6	L1C.5	5/6	0.545
10^3^					
	L9A	4/6	L1A	6/6	0.454
	L9A	4/6	L1C.5	6/6	0.454
	L1A	6/6	L1C.5	6/6	1.000
10^4^					
	L9A	6/6	L1A	6/6	1.000
	L9A	6/6	L1C.5	6/6	1.000
	L1A	6/6	L1C.5	6/6	1.000

A comment has been placed requesting assistance to add a footnote with the following * denotes a statistically significant difference (p-value 0.05)

### Median infectious dose and minimum infectious dose

The variant with the lowest ID_50_ was L1C.5 as shown in Table 3. Regarding MID at dpi 4, both L1C.5 and L1A had lower MIDs compared to the L9A variant, with MID of less than 10^1.5^ TCID_50_/mL (No cytopathic effect [NCPE]), and 10^1.75^ TCID_50_/mL, respectively.

**Table 3:** **Probit regression model results for median infectious dose (ID**_50_),** and minimum infectious dose for each PRRSV variant, in TCID**_50_/mL

PRRSV variant	ID_50_ (95% Confidence Interval)	Minimum infectious dose
L9A	10^2.6^ (10^2.0^, 10^3.2^)	10^1.8^
L1A	10^2.3^ (10^1.6^, 10^3.0^)	10^1.5^
L1C.5	10^1.3^ (10^0.4^, 10^2.2^)	10^1.5^

### Nasal and fecal viral shedding

**Figure 2:**
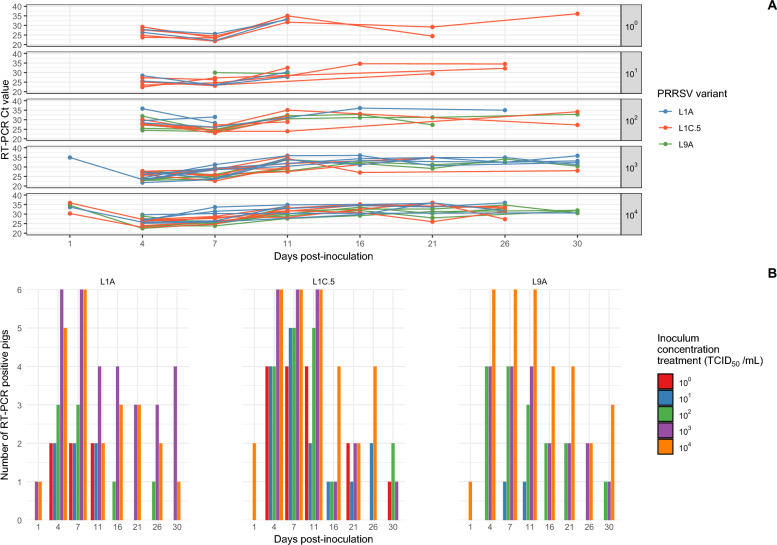
PRRSV RT-PCR Ct values and number of positive nasal swabs by inoculum concentration and variant. **A** Nasal swab PRRSV RT-PCR Ct values per virus group, inoculum concentration treatment and pig for each day post-inoculation (dpi), and **B** of PRRSV RT-PCR positive pigs using nasal swabs per virus group and inoculum concentration treatment for each day post-inoculation (dpi)

**Figure 3:**
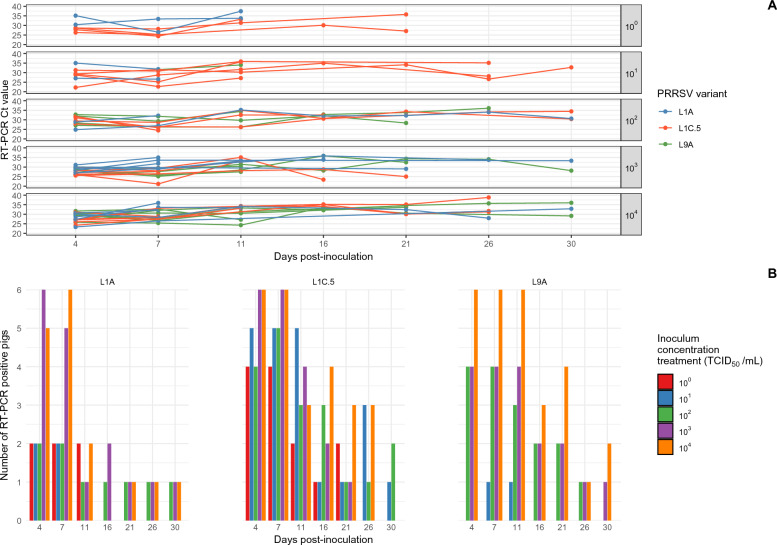
**PRRSV RT-PCR Ct values and number of positive rectal swabs by inoculum concentration and variant.** **A** Rectal swab PRRSV RT-PCR Ct values per virus group, inoculum concentration treatment and pig for each day post-inoculation (dpi), and **B** number of PRRSV RT-PCR positive pigs using rectal swabs per virus group and inoculum concentration treatment for each day post-inoculation (dpi).

Detection of PRRSV RNA in nasal swabs began at 1 dpi with 1 or 2 pigs from the highest inoculum concentration testing RT-PCR positive regardless of the PRRSV variant. The number of positive samples decreased over time across all groups in the rest of the inoculum concentration treatments, and the Ct values had an overall increase trend as the experiment progressed (Figure 2). By 7 dpi, all nasal swabs tested positive (Table 4), and nasal shedding over time varied between variants and inoculum concentrations used for the challenge (Figure 2). Similarly, PRRSV RNA in rectal swabs was detected in most pigs across all inoculum concentrations and variants by 4 dpi. By 7 dpi, all but one pig tested positive in rectal swabs (Table 5) (Figure 3). Shedding of PRRSV in feces was variable among concentration and variant groups (Figure 3).

**Table 4:** **Number of pigs that tested PRRSV positive by RT-PCR and the median Ct value in nasal swabs after inoculation by PRRSV variant and concentration of the inocula**

PRRSV variant	Concentration treatment	Sampling day after inoculation							
		1	4	7	11	16	21	26	30
L9A	10^0^	*	*	*	*	*	*	*	*
	10^1^	0/1 (ND)	0/1 (ND)	1/1 (30.0)	1/1 (29.5)	*	*	*	*
	10^2^	0/4 (ND)	4/4 (26.4)	4/4 (23.5)	3/4 (32.1)	2/2 (32.0)	2/2 (29.3)	0/2 (ND)	1/2 (32.8)
	10^3^	0/4 (ND)	4/4 (23.5)	4/4 (25.4)	4/4 (29.1)	2/2 (32.2)	2/2 (30.2)	2/2 (33.4)	1/2 (30.6)
	10^4^	1/6 (34.6)	6/6 (25.2)	6/6 (25.1)	6/6 (30.5)	4/4 (32.2)	4/4 (30.8)	2/4 (33.8)	3/4 (31.8)
L1A	10^0^	0/2 (ND)	2/2 (27.1)	2/2 (23.8)	2/2 (33.3)	*	*	*	*
	10^1^	0/2 (ND)	2/2 (27.0)	2/2 (23.5)	2/2 (29.0)	*	*	*	*
	10^2^	0/3 (ND)	3/3 (29.5)	3/3 (28.3)	0/3 (ND)	1/1 (26.1)	0/1 (ND)	1/1 (35.0)	0/1 (ND)
	10^3^	1/6 (34.9)	6/6 (23.8)	6/6 (28.9)	4/6 (34.2)	3/4 (33.8)	3/4 (34.9)	3/4 (32.4)	4/4 (32.7)
	10^4^	1/6 (33.7)	5/6 (26.6)	6/6 (30.0)	2/6 (34.6)	3/4 (34.4)	3/4 (35.7)	2/4 (33.8)	1/4 (30.6)
L1C.5	10^0^	0/4 (ND)	4/4 (26.2)	4/4 (23.5)	4/4 (34.1)	0/2 (ND)	2/2 (26.8)	0/2 (ND)	1/2 (36.1)
	10^1^	0/5 (ND)	4/5 (24.2)	5/5 (24.6)	2/5 (30.4)	1/3 (34.6)	1/3 (29.4)	2/3 (33.4)	0/3 (ND)
	10^2^	0/5 (ND)	4/5 (28.1)	5/5 (24.3)	5/5 (31.0)	1/3 (33.1)	0/3 (ND)	0/3 (ND)	2/3 (30.7)
	10^3^	0/6 (ND)	6/6 (26.4)	6/6 (27.3)	6/6 (30.9)	1/4 (27.1)	2/4 (34.7)	0/4 (ND)	1/4 (28.0)
	10^4^	2/6 (33.1)	6/6 (25.7)	6/6 (27.9)	6/6 (31.7)	4/4 (33.6)	2/4 (31.0)	4/4 (32.3)	0/3 (ND)

ND = no detection, * = no PRRSV-positive pigs by RT-PCR in serum samples

**Table 5:** **Number of pigs that tested PRRSV positive by RT-PCR and the median Ct value in rectal swabs after inoculation by PRRSV variant and concentration of the inocula**

PRRSV variant	Concentration treatment	Sampling day after inoculation							
		1	4	7	11	16	21	26	30
L9A	10^0^	*	*	*	*	*	*	*	*
	10^1^	0/1 (ND)	0/1 (ND)	1/1 (34.6)	1/1 (34.0)	*	*	*	*
	10^2^	0/4 (ND)	4/4 (30.2)	4/4 (27.9)	3/4 (29.7)	2/2 (32.5)	2/2 (31.1)	1/2 (36.1)	0/2 (ND)
	10^3^	0/4 (ND)	4/4 (28.5)	4/4 (26.7)	4/4 (29.4)	2/2 (32.0)	2/2 (33.4)	1/2 (34.1)	1/2 (28.1)
	10^4^	0/6 (ND)	6/6 (29.5)	6/6 (29.1)	6/6 (30.9)	3/4 (32.4)	4/4 (34.0)	1/4 (35.7)	2/4 (32.6)
L1A	10^0^	0/2 (ND)	2/2 (32.8)	2/2 (29.9)	2/2 (35.6)	*	*	*	*
	10^1^	0/2 (ND)	2/2 (31.0)	2/2 (29.2)	0/2 (ND)	*	*	*	*
	10^2^	0/3 (ND)	2/3 (26.9)	2/3 (29.6)	1/3 (35.2)	1/1 (32.1)	1/1 (32.3)	1/1 (34.1)	1/1 (30.7)
	10^3^	0/6 (ND)	6/6 (28.8)	5/6 (31.8)	1/6 (33.2)	2/4 (34.9)	1/4 (29.0)	1/4 (33.8)	0/4 (ND)
	10^4^	0/6 (ND)	5/6 (28.6)	6/6 (32.4)	2/6 (33.7)	0/4 (ND)	1/4 (32.4)	1/4 (28.0)	1/4 (32.9)
L1C.5	10^0^	0/4 (ND)	4/4 (28.2)	4/4 (25.2)	2/4 (32.3)	1/2 (30.1)	2/2 (31.4)	0/2 (ND)	0/2 (ND)
	10^1^	0/5 (ND)	5/5 (29.3)	5/5 (28.8)	5/5 (31.7)	1/3 (34.9)	1/3 (34.1)	3/3 (28.1)	1/3 (32.8)
	10^2^	0/5 (ND)	4/5 (30.2)	5/5 (26.2)	3/5 (32.6)	3/3 (30.8)	1/3 (34.3)	1/3 (34.1)	2/3 (32.4)
	10^3^	0/6 (ND)	6/6 (26.7)	6/6 (27.0)	4/6 (33.3)	2/4 (26.1)	1/4 (25.0)	0/4 (ND)	0/4 (ND)
	10^4^	0/6 (ND)	6/6 (27.1)	6/6 (28.2)	3/6 (34.1)	4/4 (35.1)	3/4 (30.1)	3/4 (31.7)	0/3 (ND)

ND = no detection, * = no PRRSV-positive pigs by RT-PCR in serum samples

### Histopathology

The most common tissue with lesions across all three variants was the lung (20/30 pigs), followed by the heart (8/30 pigs) and the brain (7/30 pigs). Overall, lung findings included mild interstitial pneumonia (focal or multifocal, necrotic, with or without lymphocytic perivasculitis), bronchopneumonia, catarrhal bronchitis, mild lymphoplasmacytic alveolitis, and perivasculitis across all inoculum concentrations of the three variants. Heart lesions primarily consisted of mild to marked lymphocytic or lymphoplasmacytic interstitial myocarditis, and focal lymphocytic epicarditis. Brain lesions included choroid plexus’ capillaries thinly cuffed by lymphocytes, and scattered blood vessels thinly cuffed by lymphocytes. In the liver, lesions included slight to mild perilobular and portal lymphohistiocytic hepatitis. Tonsil germinal centers were expanded by blast-transformed lymphocytes, defined as having germinal center hypertrophy and hyperplasia. Lymph nodes also had germinal centers expanded by blast-transformed lymphocytes with partial loss of normal architecture as well as areas of medullary edema containing scattered polykaryocytes. Some lymph nodes contained scattered small numbers of neutrophils.

For L9A, lesions were found in one out of 10 brains, four out of 10 lungs, one had liver lesions, and three lesions in the bronchial lymph node. Unexpectedly, both pigs in the control treatment also presented mild multifocal interstitial pneumonia or just interstitial pneumonia and one pig had a lesion in the bronchial lymph node that was blast-active with partial loss of normal architecture. For L1A, lesions were found in one of the 10 brains, six out of 10 lungs, one out of 10 hearts, one out of 10 superficial inguinal lymph nodes. One pig also had lesions in the medial iliac lymph node. In the control treatment, one out of six presented lesions in both the brain and the lung, described as scattered blood vessels thinly cuffed by lymphocytes, which are non-specific to PRRSV.

Regarding the variant L1C.5, seven out of 10 pigs had brain lesions, 10 out of 10 had lung lesions, seven out of 10 had heart lesions, one out of 10 had lesions in the tonsil, two out of 10 had lesions in the superficial inguinal lymph node, one out 10 had lesions in the retropharyngeal lymph node, one out of 10 had lesions in the medial iliac lymph node, one out of 10 had lesions in the thoracic lymph node, three out of 10 had lesions in the bronchial lymph node. Control pigs did not present any lesions. Findings are summarized in Tables 6 and 7.

**Table 6:** **Tissues with histopathological findings by PRRSV variant and inoculum concentration treatment at day 11 post-inoculation**

PRRSV variant	Inoculum concentration treatment	Pig	Tissue sample									
			Brain	Lung	Heart	Liver	Tonsil	Lymph node				
								Superficial inguinal	Retropharyngeal	Medial iliac	Thoracic	Bronchial
L9A	Control	1		X								
		2		X								X
	10^0^	1										
		2										
	10^1^	1										
		2										
	10^2^	1				X						
		2	X	X								
	10^3^	1		X								X
		2		X								X
	10^4^	1		X								X
		2										
L1A	Control	1										
		2	X	X								
	10^0^	1		X								
		2		X				X				
	10^1^	1		X								
		2		X						X		
	10^2^	1										
		2		X								
	10^3^	1										
		2										
	10^4^	1										
		2	X	X	X							
L1C.5	Control	1										
		2										
	10^0^	1		X	X		X		X			X
		2	X	X	X			X				
	10^1^	1	X	X	X							X
		2	X	X								
	10^2^	1	X	X	X							
		2		X	X							
	10^3^	1		X	X							
		2	X	X								
	10^4^	1	X	X	X			X		X	X	X
		2	X	X

**Table 7:** **Histopathology results by PRRSV variant, inoculum concentration treatment, pig, and tissue**

PRRSV variant	Concentration group	Pig ID	Tissue sample	Histopathologic result
L9A	Control	1215	Lung	Interstitial pneumonia, mild, multifocal
		1224	Lung	Interstitial pneumonia
			Bronchial L.N	Blast-active, partial loss of normal architecture
	10^2^	1206	Liver	Hepatitis, perilobular and portal, lymphohistiocytic, mild/slight
		1207	Lung	Bronchopneumonia, acute, lymphocytic perivasculitis
	10^3^	1213	Lung	Bronchopneumonia, acute, lymphocytic perivasculitis
			Bronchial L.N	Mild acute lymphadenitis
		1228	Lung	Interstitial pneumonia, necrotic
			Bronchial L.N	Blast-active, partial loss of normal architecture
	10^4^	1230	Lung	Bronchopneumonia, acute, necrotic
			Bronchial L.N	Blast-active, partial loss of normal architecture
L1A	Control	782	Brain	Scattered blood vessels thinly cuffed by lymphocytes
			Lung	Scattered blood vessels thinly cuffed by lymphocytes
	10^0^	761	Lung	Interstitial pneumonia, necrotic, focal, mild
		772	Lung	Interstitial pneumonia, multifocal, mild
			Superficial inguinal L.N	Mild edema, scattered/rare polykaryocytes
	10^1^	791	Lung	Interstitial pneumonia, multifocal, mild
		805	Lung	Interstitial pneumonia, multifocal, mild; mild acute bronchitis
			Medial iliac L.N	Edema, scattered polykaryocytes
	10^2^	804	Lung	Interstitial pneumonia, necrotic, focal, mild
	10^4^	798	Lung	Interstitial pneumonia, mild
LIC.5	10^0^	1494	Lung	Interstitial pneumonia, necrotic, lymphocytic perivasculitis
			Heart	Blast-transformed
			Tonsil	Blast-transformed
			Retropharyngeal L.N	Blast-transformed
			Bronchial L.N	Blast-transformed
		1498	Brain	Scattered blood vessels thinly cuffed by lymphocytes
			Lung	Interstitial pneumonia, necrotic, lymphocytic perivasculitis
			Heart	Myocarditis, lymphocytic, mild
			Superficial inguinal L.N	Blast-transformed
	10^1^	1500	Brain	Blood vessels thinly cuffed by lymphocytes
			Lung	Bronchitis, acute, catarrhal, mild, lymphocytic perivasculitis
			Heart	Endo and myocarditis, lymphocytic, mild
			Bronchial L.N	Lymphadenitis, acute
		1522	Brain	Scattered blood vessels thinly cuffed by lymphocytes
			Lung	Interstitial pneumonia, necrotic
	10^2^	1508	Brain	Scattered blood vessels thinly cuffed by lymphocytes
			Lung	Lymphocytic perivasculitis, mild
			Heart	Myocarditis, lymphocytic, mild
		1523	Lung	Lymphocytic perivasculitis
			Heart	Myocarditis, lymphoplasmacytic, moderate
	10^3^	1511	Lung	Focal lymphocytic myocarditis/epicarditis
			Heart	Focal lymphocytic myocarditis/epicarditis
		1520	Brain	Scattered blood vessels thinly cuffed by lymphocytes
			Lung	Bronchopneumonia, necrotic, lymphocytic peribronchitis
	10^4^	1499	Brain	Scattered blood vessels thinly cuffed by lymphocytes
			Lung	Interstitial pneumonia, necrotic, lymphocytic perivasculitis
			Heart	Myocarditis, lymphocytic, moderate to marked
			Superficial inguinal L.N	Blast-transformed
			Medial iliac L.N	Blast-transformed
			Thoracic L.N	Blast-transformed
			Bronchial L.N	Blast-transformed
		1524	Brain	Scattered blood vessels thinly cuffed by lymphocytes
			Lung	Mild acute catarrhal bronchitis; mild lymphoplasmacytic alveolitis and perivasculitis

L.N. = Lymph node.

## Discussion

Through an experimental study, we were able to estimate the ID_50_ and MID of three PRRSV variants (i.e., L1C.5, L1A, L9A), describe the nasal and rectal shedding, and microscopic lesions at necropsy. Our results show that there are numerical infectivity level differences among PRRSV variants. Specifically, the L1C.5 variant needed fewer infectious virions to infect pigs, followed by the L1A and L9A variants, with a statistical difference between the overall number of positive pigs at 4 dpi between the L1C.5 both L1A and L9A, but not between L1A and L9A variants. Similar experiments to the one reported here have been conducted to assess infectiousness using different swine viruses named Senecavirus A (SVA) [32], porcine epidemic diarrhea virus (PEDV) [33], and porcine circovirus type 2 (PCV2) [34]; however, these experiments did not address differences across viral variants as diversity is much lower than PRRSV which makes our study unique. Furthermore, previous work has assessed how different routes of exposure of the same PRRSV inoculum concentration played a role in infection concluding that the ID_50_ was lower for the nasal route when compared to oral inoculation [19]. In our experiment 4 dpi was chosen as the day to consider pigs as successfully infected through inoculation. Even though biosecurity measures were kept in place pigs that became infected after 4 dpi could have been a product of indirect transmission.

Although the estimated ID_50_ differed among variants, there appears to be no differences in the nasal and fecal shedding patterns of the variants tested in this study. However, our sample size might have limited our capacity to detect such differences and our asynchronous approach makes comparisons among groups more difficult due to variations over time. Still, early presence of the virus in nasal secretions occurred in the high-concentration groups can potentially be explained by remnants of the virus from the inoculum still present on the inner nostril’s surface. Shedding via the nasal or fecal routes varied across inoculum concentrations and variants which suggests that viruses have different degrees of replication and pathogenicity [20]. Shedding was intermittent; some pigs were not consistently shedding during all sampling periods in either nasal or fecal samples, which agrees with results from previous experiments that assessed these excretion routes [13, 35–37]. The inconsistent detection of the viruses could be attributed to factors other than the host not shedding, such as the replication potential and virulence of different PRRS viruses in the nasal mucosa and this being underestimated by the instrument of collection [38, 39]. For nasal and fecal samples, rayon tipped swabs were used which can have the disadvantage of having a tight fiber structure that can trap the sample hindering its release. The pathogen detection performance of the swab tip material may depend on the pathogen and the host, for example, when compared to nylon swabs for the detection of respiratory bacteria in nasopharyngeal carriage in children there appears to be no statistical difference in the detection [40], and for Influenza A virus (IAV) detection it is mentioned more viral particles are detected in polyester swabs than cotton swabs [41].

The number of lesions found through the histopathologic assessment varied among variants, inoculum concentration, and tissue. Pulmonary lesions were the most common finding across the three variants, characterized by interstitial pneumonia, whether it was focal or multifocal, necrotic, with or without lymphocytic perivasculitis. Previous experiments [37, 42, 43] in which pigs were exposed intranasally using a concentration of 10^5^ TCID_50_/mL to 10^5.8^ TCID_50_/mL doses of PRRSV (i.e., ATCC VR-2332, SNUVR090851) had similar lesions as interstitial pneumonia, infiltration of mononuclear cells in alveoli, bronchopneumonia, and alveolar exudate were reported. Although this lesion was found during the microscopic examination in the inoculated pigs, two of the control pigs from the L9A variant cohort also had this lesion. This type of lesion is not pathognomonic of PRRSV as it has been described in pigs exposed to other agents such as porcine respiratory coronavirus (PRCV), PCV2, PCV3, *Escherichia coli*, and ammonia [44–48]. These control pigs were tested for PRRSV and confirmed that they were negative during the post-mortem examination which rules out this virus as the causative agent of the interstitial pneumonia. Cardiac and brain lesions from our study had similar descriptions from previous work [13, 43, 49] but they were more consistently found in pigs infected with the L1C.5 variant group. Rawal et al. found that all but one of the L1 isolates used induced neurological clinical signs and brain infection [22]. From our study one pig from L9A and another from L1A groups had brain lesions, whereas in the L1C.5 group, at least one pig from each concentration exhibited lesions. Heart lesions were mostly found in L1C.5 group pigs. These findings suggest that the L1C.5 variant can affect tissues not commonly affected by other variants. All ID_50_, nasal and rectal viral detection, and tissue lesions could have been affected from the difference in number of cell culture passages of each variant, as L1C.5 had less than L1A and L9A. Studies on the effect of the number of passages on virulence have been conducted using PEDV and porcine deltacoronavirus (PDCoV) [50, 51]. These studies reported that there can be changes in virulence when assessing shedding, clinical signs, and histological lesions, but in the case of PDCoV low-passage variants produced less obvious CPEs, although the number of serial passages described in both these studies is greater than the ones used in our study.

In the present study, we hypothesized that PRRSV variants would require differing amounts of infective units to begin infection in weaned pigs after intranasal inoculation. Additionally, the variant that needed fewer viral particles also showed a more distinct fecal and nasal shedding pattern, and caused more microscopic lesions in collected tissues. This study incorporates the estimation of the MID and ID_50_ using five different inoculum concentrations for three different PRRSV variants, from which we conclude that one of these three variants (i.e., L1C.5) can cause infection with fewer viral particles, and microscopic lesions appear to be present in more tissues, but the fecal and nasal shedding patterns appear to be similar to the other two variants (i.e., L1A, L9A). As we used only one route of inoculation, future studies could incorporate not only different inoculum concentrations and variants, but also other routes of inoculation. Moreover, oral swabs could be incorporated to assess oral shedding to compare shedding patterns to the ones described in our study.

## Data Availability

The datasets used and/or analyzed during the current study are available from the corresponding author upon reasonable request.

## References

[1] Kuhn JH, Lauck M, Bailey AL, Shchetinin AM, Vishnevskaya TV, Bào Y, Ng TFF, LeBreton M, Schneider BS, Gillis A, Tamoufe U, Diffo JLD, Takuo JM, Kondov NO, Coffey LL, Wolfe ND, Delwart E, Clawson AN, Postnikova E, Bollinger L, Lackemeyer MG, Radoshitzky SR, Palacios G, Wada J, Shevtsova ZV, Jahrling PB, Lapin BA, Deriabin PG, Dunowska M, Alkhovsky SV, Rogers J, Friedrich TC, O’Connor DH, Goldberg TL (2016) Reorganization and expansion of the nidoviral family *Arteriviridae*. Arch Virol 161:755–768 26608064 10.1007/s00705-015-2672-z PMC5573231

[2] Zerbini FM, Siddell SG, Lefkowitz EJ, Mushegian AR, Adriaenssens EM, Alfenas-Zerbini P, Dempsey DM, Dutilh BE, Garcia ML, Hendrickson RC, Junglen S, Krupovic M, Kuhn JH, Lambert AJ, Lobocka M, Oksanen HM, Robertson DL, Rubino L, Sabanadzovic S, Simmonds P, Smith DB, Suzuki N, Van Doorslaer K, Vandamme AM, Varsani A (2023) Changes to virus taxonomy and the ICTV statutes ratified by the international committee on taxonomy of viruses. Arch Virol 168:175 37296227 10.1007/s00705-023-05797-4 PMC10861154

[3] Osemeke O, Corzo CA, Kikuti M, Yue X, Vadnais S, Silva G, Linhares D, Holtkamp D (2024) Updates on the economic impact of PRRSV to US pork producers. In: 2024 Allen D Leman Swine Conference Research Abstracts and Proceedings, Saint Paul, Minnesota, USA, September 2024. University of Minnesota, pp 174

[4] Fraile L (2012) Control or eradication? Costs and benefits in the case of PRRSV. Vet Rec 170:223–224 22391905 10.1136/vr.e1386

[5] Batista L, Dee SA, Rossow KD, Deen J, Pijoan C (2002) Assessing the duration of persistence and shedding of porcine reproductive and respiratory syndrome virus in a large population of breeding-age gilts. Can J Vet Res 66:196–200 12146892 PMC227004

[6] Díaz I, Gimeno M, Darwich L, Navarro N, Kuzemtseva L, López S, Galindo I, Segalés J, Martín M, Pujols J, Mateu E (2012) Characterization of homologous and heterologous adaptive immune responses in porcine reproductive and respiratory syndrome virus infection. Vet Res 43:30 22515169 10.1186/1297-9716-43-30 PMC3403850

[7] Guo R, Shang P, Carrillo CA, Sun Z, Lakshmanappa YS, Yan X, Renukaradhya GJ, McGill J, Jaing CJ, Niederwerder MC, Rowland RRR, Fang Y (2018) Double-stranded viral RNA persists in vitro and in vivo during prolonged infection of porcine reproductive and respiratory syndrome virus. Virology 524:78–89 30165309 10.1016/j.virol.2018.08.006

[8] Horter DC, Pogranichniy RM, Chang CC, Evans RB, Yoon KJ, Zimmerman JJ (2002) Characterization of the carrier state in porcine reproductive and respiratory syndrome virus infection. Vet Microbiol 86:213–228 11900956 10.1016/s0378-1135(02)00013-5

[9] Wills RW, Doster AR, Galeota JA, Sur JH, Osorio FA (2003) Duration of infection and proportion of pigs persistently infected with porcine reproductive and respiratory syndrome virus. J Clin Microbiol 41:58–62 12517825 10.1128/JCM.41.1.58-62.2003 PMC149563

[10] Klinge KL, Vaughn EM, Roof MB, Bautista EM, Murtaugh MP (2009) Age-dependent resistance to porcine reproductive and respiratory syndrome virus replication in swine. Virol J 6:177 19860914 10.1186/1743-422X-6-177 PMC2773768

[11] Music N, Gagnon CA (2010) The role of porcine reproductive and respiratory syndrome (PRRS) virus structural and non-structural proteins in virus pathogenesis. Anim Health Res Rev 11:135–163 20388230 10.1017/S1466252310000034

[12] Pileri E, Mateu E (2016) Review on the transmission porcine reproductive and respiratory syndrome virus between pigs and farms and impact on vaccination. Vet Res 47:108 27793195 10.1186/s13567-016-0391-4 PMC5086057

[13] Rossow KD, Bautista EM, Goyal SM, Molitor TW, Murtaugh MP, Morrison RB, Benfield DA, Collins JE (1994) Experimental porcine reproductive and respiratory syndrome virus infection in one-, four-, and 10-week-old pigs. J Vet Diagn Invest 6:3–12 8011777 10.1177/104063879400600102

[14] Rossow KD, Collins JE, Goyal SM, Nelson EA, Christopher-Hennings J, Benfield DA (1995) Pathogenesis of porcine reproductive and respiratory syndrome virus infection in gnotobiotic pigs. Vet Pathol 32:361–373 7483210 10.1177/030098589503200404

[15] Zimmerman JJ, Karriker, Locke A., Ramirez A, Schwartz KJ, Stevenson GW (eds) (2012) Diseases of Swine. 10th ed. Wiley-Blackwell, Ames

[16] Christianson WT, Choi CS, Collins JE, Molitor TW, Morrison RB, Joo HS (1993) Pathogenesis of porcine reproductive and respiratory syndrome virus infection in mid-gestation sows and fetuses. Can J Vet Res 57:262–268 8269364 PMC1263638

[17] Dee S, Deen J, Rossow K, Weise C, Eliason R, Otake S, Joo HS, Pijoan C (2003) Mechanical transmission of porcine reproductive and respiratory syndrome virus throughout a coordinated sequence of events during warm weather. Can J Vet Res 67:12–19 12528824 PMC227022

[18] Otake S, Dee SA, Rossow KD, Deen J, Joo HS, Molitor TW, Pijoan C (2002) Transmission of porcine reproductive and respiratory syndrome virus by fomites (boots and coveralls). J Swine Health Prod 10:59–65

[19] Hermann JR, Muñoz-Zanzi CA, Roof MB, Burkhart K, Zimmerman JJ (2005) Probability of porcine reproductive and respiratory syndrome (PRRS) virus infection as a function of exposure route and dose. Vet Microbiol 110:7–16 16098692 10.1016/j.vetmic.2005.06.012

[20] Ruedas-Torres I, Rodríguez-Gómez IM, Sánchez-Carvajal JM, Larenas-Muñoz F, Pallarés FJ, Carrasco L, Gómez-Laguna J (2021) The jigsaw of PRRSV virulence. Vet Microbiol 260:109168 34246042 10.1016/j.vetmic.2021.109168

[21] Kikuti M, Paploski IAD, Pamornchainavakul N, Picasso-Risso C, Schwartz M, Yeske P, Leuwerke B, Bruner L, Murray D, Roggow BD, Thomas P, Feldmann L, Allerson M, Hensch M, Bauman T, Sexton B, Rovira A, VanderWaal K, Corzo CA (2021) Emergence of a new lineage 1C variant of porcine reproductive and respiratory syndrome virus 2 in the United States. Front Vet Sci 8:752938 34733906 10.3389/fvets.2021.752938 PMC8558496

[22] Rawal G, Almeida MN, Gauger PC, Zimmerman JJ, Ye F, Rademacher CJ, Armenta Leyva B, Munguia-Ramirez B, Tarasiuk G, Schumacher LL, Aljets EK, Thomas JT, Zhu JH, Trexel JB, Zhang J (2023) In vivo and In vitro characterization of the recently emergent PRRSV 1-4-4 L1C variant (L1C.5) in comparison with other PRRSV-2 lineage 1 isolates. Viruses 15:2233 38005910 10.3390/v15112233 PMC10674456

[23] VanderWaal K, Pamornchainavakul N, Kikuti M, Linhares DCL, Trevisan G, Zhang J, Anderson TK, Zeller M, Rossow S, Holtkamp DJ, Makau DN, Corzo CA, Paploski IAD (2024) Phylogenetic-based methods for fine-scale classification of PRRSV-2 ORF5 sequences: a comparison of their robustness and reproducibility. Front Virol 4:1433931

[24] Paploski IAD, Pamornchainavakul N, Makau DN, Rovira A, Corzo CA, Schroeder DC, Cheeran MC-J, Doeschl-Wilson A, Kao RR, Lycett S, VanderWaal K (2021) Phylogenetic structure and sequential dominance of sub-lineages of PRRSV type-2 lineage 1 in the United States. Vaccines 9:608 34198904 10.3390/vaccines9060608 PMC8229766

[25] Shi M, Lam TTY, Hon CC, Murtaugh MP, Davies PR, Hui RKH, Li J, Wong LTW, Yip CW, Jiang JW, Leung FCC (2010) Phylogeny-based evolutionary, demographical, and geographical dissection of North American type 2 porcine reproductive and respiratory syndrome viruses. J Virol 84:8700–8711 20554771 10.1128/JVI.02551-09 PMC2919017

[26] Yim-im W, Anderson TK, Paploski IAD, VanderWaal K, Gauger P, Krueger K, Shi M, Main R, Zhang J (2023) Refining PRRSV-2 genetic classification based on global ORF5 sequences and investigation of their geographic distributions and temporal changes. Microbiol Spectr 11:e02916-e2923 37933982 10.1128/spectrum.02916-23 PMC10848785

[27] Kim HS, Kwang J, Yoon IJ, Joo HS, Frey ML (1993) Enhanced replication of porcine reproductive and respiratory syndrome (PRRS) virus in a homogeneous subpopulation of MA-104 cell line. Arch Virol 133:477–483 8257302 10.1007/BF01313785

[28] Ramakrishnan MA (2016) Determination of 50% endpoint titer using a simple formula. World J Virol 5:85–86 27175354 10.5501/wjv.v5.i2.85 PMC4861875

[29] Venables WN, Ripley BD (2002) Modern applied statistics with S, 4th edn. Springer, New York

[30] R Core Team (2024) R: a language and environment for statistical computing. Vienna. https://r-project.org/. Accessed 8 Jul 2025

[31] Dunn PK, Smyth GK (2018) Generalized linear models with examples in R. Springer, New York

[32] Buckley A, Lager K (2022) Infectious dose of Senecavirus A in market weight and neonatal pigs. PLoS One 17:e0267145 35486625 10.1371/journal.pone.0267145 PMC9053780

[33] Schumacher LL, Woodworth JC, Jones CK, Chen Q, Zhang J, Gauger PC, Stark CR, Main RG, Hesse RA, Tokach MD, Dritz SS (2016) Evaluation of the minimum infectious dose of porcine epidemic diarrhea virus in virus-inoculated feed. Am J Vet Res 77:1108–1113 27668582 10.2460/ajvr.77.10.1108

[34] Tomás A, Fernandes LT, Valero O, Segalés J (2008) A meta-analysis on experimental infections with porcine circovirus type 2 (PCV2). Vet Microbiol 132:260–273 18614300 10.1016/j.vetmic.2008.05.023

[35] Wills RW, Zimmerman JJ, Yoon KJ, Swenson SL, McGinley MJ, Hill HT, Platt KB, Christopher-Hennings J, Nelson EA (1997) Porcine reproductive and respiratory syndrome virus: a persistent infection. Vet Microbiol 55:231–240 9220618 10.1016/s0378-1135(96)01337-5

[36] Opriessnig T, Rawal G, McKeen L, Filippsen Favaro P, Halbur PG, Gauger PC (2021) Evaluation of the intranasal route for porcine reproductive and respiratory disease modified-live virus vaccination. Vaccine 39:6852–6859 34706840 10.1016/j.vaccine.2021.10.033

[37] Park C, Seo HW, Han K, Kang I, Chae C (2014) Evaluation of the efficacy of a new modified live porcine reproductive and respiratory syndrome virus (PRRSV) vaccine (Fostera PRRS) against heterologous PRRSV challenge. Vet Microbiol 172:432–442 24970363 10.1016/j.vetmic.2014.05.030

[38] Frydas IS, Nauwynck HJ (2016) Replication characteristics of eight virulent and two attenuated genotype 1 and 2 porcine reproductive and respiratory syndrome virus (PRRSV) strains in nasal mucosa explants. Vet Microbiol 182:156–162 26711043 10.1016/j.vetmic.2015.11.016

[39] Martín-Valls GE, Li Y, Díaz I, Cano E, Sosa-Portugal S, Mateu E (2022) Diversity of respiratory viruses present in nasal swabs under influenza suspicion in respiratory disease cases of weaned pigs. Front Vet Sci 19:1014475 10.3389/fvets.2022.1014475 PMC9627340 36337208

[40] Wigger C, Morris PS, Stevens M, Smith-Vaughan H, Hare K, Beissbarth J, Leach AJ (2019) A comparison of flocked nylon swabs and non-flocked rayon swabs for detection of respiratory bacteria in nasopharyngeal carriage in Australian Indigenous children. J Microbiol Methods 157:47–49 30578888 10.1016/j.mimet.2018.12.013

[41] Turlewicz-Podbielska H, Włodarek J, Pomorska-Mól M (2020) Noninvasive strategies for surveillance of swine viral diseases: a review. J Vet Diagn Invest 32:503–512 32687007 10.1177/1040638720936616 PMC7438649

[42] Darbès J, Hafner A, Breuer W, Hänichen T, Banholzer E, Hermanns W (1996) Histopathological findings in pigs of various ages suffering from spontaneous infection with the porcine reproductive and respiratory syndrome virus (PRRSV). Zentralbl Veterinarmed A 43:353–363 8818300 10.1111/j.1439-0442.1996.tb00463.x

[43] Halbur PG, Paul PS, Meng XJ, Lum MA, Andrews JJ, Rathje JA (1996) Comparative pathogenicity of nine US porcine reproductive and respiratory syndrome virus (PRRSV) isolates in a five-week-old cesarean-derived, colostrum-deprived pig model. J Vet Diagn Invest 8:11–20 9026065 10.1177/104063879600800103

[44] Janke BH (1995) Diagnosis of viral respiratory disease in swine. Swine Health Prod 3:116–120

[45] Sarli G, D’Annunzio G, Gobbo F, Benazzi C, Ostanello F (2021) The role of pathology in the diagnosis of swine respiratory disease. Vet Sci 8:256 34822629 10.3390/vetsci8110256 PMC8618091

[46] Oh B, Park J, Kim E, Seo S, Kim B, Oh SI (2024) Alteration of growth performance and characterization of pathological lesions in long-term ammonia-exposed pigs. Ecotoxicol Environ Saf 287:117318 39536557 10.1016/j.ecoenv.2024.117318

[47] Wray C, Piercy DWT, Carroll PJ, Cooley WA (1993) Experimental infection of neonatal pigs with CNF toxin-producing strains of Escherichia coli. Res Vet Sci 54:290–298 8337478 10.1016/0034-5288(93)90125-y

[48] Molossi FA, De Almeida BA, De Cecco BS, Da Silva MS, Mósena ACS, Brandalise L, Simão GMR, Canal CW, Vanucci F, Pavarini SP, Driemeier D (2022) A putative PCV3-associated disease in piglets from Southern Brazil. Braz J Microbiol 53:491–498 34988935 10.1007/s42770-021-00644-7 PMC8882493

[49] Papatsiros V, Stylianaki I, Papakonstantinou G, Tsekouras N, Bitchava D, Christodoulopoulos G, Papaioannou N (2020) Histopathological lesions accompanied with first-time isolation of a PRRSV-2 strain in Greece. Viral Immunol 33:565–570 33001795 10.1089/vim.2020.0087

[50] Lin CM, Hou Y, Marthaler DG, Gao X, Liu X, Zheng L, Saif LJ, Wang Q (2017) Attenuation of an original US porcine epidemic diarrhea virus strain PC22A via serial cell culture passage. Vet Microbiol 201:62–71 28284624 10.1016/j.vetmic.2017.01.015 PMC7117544

[51] Zhang L, Yu R, Wang L, Zhang Z, Lu Y, Zhou P, Wang Y, Guo H, Pan L, Liu X (2024) Serial cell culture passaging *in vitro* led to complete attenuation and changes in the characteristic features of a virulent porcine deltacoronavirus strain. J Virol 98:e0064524 39012141 10.1128/jvi.00645-24 PMC11334472

